# Meeting September 17

**Published:** 1886-11

**Authors:** 

**Affiliations:** 2330 Indiana Avenue


W. W. JAGGARD, M.D., *Editor.*

2330 Indiana Avenue, October 1886.

---

### CHICAGO GYNÆCOLOGICAL SOCIETY.

*Regular Meeting, September 17, 1886.*—THE PRESIDENT, DANIEL T. NELSON, M.D., in the chair.

*I.*—ETHERIDGE. *Report of a case of Supra-Vaginal Amputation of the Pregnant Uterus, Complicating a Multilocular Fibroid Tumor.*

*II.*—PARKES. *Successful Removal of the Uterus for Fibroids.*

PROFESSOR JAMES H. ETHERIDGE read a paper entitled

### I.—REPORT OF A CASE OF SUPRA-VAGINAL AMPUTATION OF THE PREGNANT UTERUS, COMPLICATING A MULTILOCULAR FIBROID TUMOR.

Mrs. A. B., aged 34 years, married several years, no children, first experienced uterine symptoms some four years ago, which Dr. Knox, her medical attendant, recognized as due to a uterine retroversion, and treated with a pessary for six months. On March 10, 1884, he was again called to her, and found the uterus again retroverted. "This time, however," he writes, "it was apparently fixed in the pelvis; the *os tincæ* was caught firmly behind the pubes, and in Douglas's *cul-de-sac* was a firm sensitive mass that seemed to be more than the *fundus uteri.* There was also considerable abdominal tenderness, fever, and nausea. It was difficult to diagnosticate between cellulitis and simple retroversion with impaction. Careful manipulation, however, in Sims's position, corrected the displacement with complete relief of the distressing symptoms. Hot fomentations and vaginal douches aborted the threatening cellulitis. Two days thereafter, on careful examination, I detected a subperitoneal myoma on the left posterior aspect of the fundus. It was about half the size of an unimpregnated uterus. This accounted for the tight impaction of the retroverted organ.

"A pessary was again introduced, and twenty minim doses fluid extract of ergot, three times a day, ordered. The drug was faithfully taken for about twelve months. During that time a second fibroid appeared upon the right lower and anterior surface of the womb. These tumors steadily enlarged until the uterus was lifted entirely out of the pelvis. Excepting the results of mechanical pressure, the patient's health was excellent, and menstruation was undisturbed.

"April, 1885, the ergot was discontinued. Owing to the sub-peritoneal character of the fibromata, their growth was uninfluenced by the drug."

The growth of the tumor in the last two years has been slow but progressive. In the last six months it caused no special symptoms beyond vague supra-pubic pains at times.

In May, 1886, thirteen months after the stopping of the use of ergot, Dr. Knox was again summoned to attend her for a distressing nausea. At the same time the menses, which had never been excessive, ceased. Mammary changes supervened.

In the ensuing three months the tumor grew rapidly. To Dr. Knox must be ascribed diagnostic skill of the highest order in determining the presence of pregnancy in such a mystifying condition of things. He decided that she must be pregnant, and awaited the expiration of the first three months to produce an abortion, hoping thereby to induce involution to such an extent as, at least, to arrest the rapid increase in the growth of the tumor. Accordingly, on August 1, 1886, when the three months had expired, he introduced a sound into the uterus four inches. Its withdrawal was followed by a small amount of blood, the nausea and vomiting ceased, and the mammary symptoms disappeared. Nothing further ever followed indicating the previous existence of pregnancy or an abortion, and the conclusion was reached that conception had not occurred.

The rapid encroachment on the abdominal organs was progressively killing her. Her strength had greatly diminished. For six months she gradually emaciated. Her sufferings from the pressure of the tumor were great, and eventually led to her giving up and remaining in bed nearly all the time, because she thus experienced the most comfort. In addition she was easily put out of breath by exertion.

Forty hours before the operation she had a free purge, and twenty hours later she went to the Presbyterian Hospital to remain overnight, to receive a bichloride of mercury bath and to have her pubes shaved.

At the time of the operation she presented the following measurements:

1. Girth at the umbilicus, 31 inches.
2. From ensiform cartilage to umbilicus, 7 inches.
3. From umbilicus to symphysis pubis, 6.5 inches.
4. From either ant. sup. spin. proc. to umbilicus, 7 inches.

From external examination it was found that the tumor extended from the right iliac fossa across the abdominal cavity in a straight line to the spleen. Its length was, apparently, double or treble its width. It was freely movable, free from adhesions, and solid. It presented great tenderness in the right iliac fossa.

*Per vaginam* the *cervix uteri* was found very high up in the left iliac fossa and the *fundus uteri* was apparently thrust into the right iliac region. The whole tumor moved with the uterus. A very small resiliency offered to conjoined manipulation led me to think that I had to do with a *fibro-cystic tumor of the uterus.* The sound entered the uterus four inches and seemed to pass towards the umbilicus.

Just before commencing the operation a sound introduced into the bladder showed that this viscus was not enlarged by being drawn up out of the pelvis by the growth of the tumor.

At the same time the sound was introduced into the uterine cavity and entered only four inches. It had to be bent at an obtuse angle to make it engage in the cervical canal. Its introduction was attended with a small hæmorrhage, which subsequently was the innocent cause of severe but brief alarm.

The instruments used in the operation were dipped in a five per centum solution of carbolic acid, and subsequently kept in a two per centum solution of the same agent when not in actual use. No spray was used. The sponges were antisepticised with carbolic acid. The details of the operation were simplicity itself. The incision began one inch above the umbilicus and extended down to within an inch and a half above the pubes. It was carried to the left of the umbilicus and measured six inches. Upon exposing the tumor it was found wholly free from adhesions. It extended to, and pressed upwards, the spleen. It was oblong, its length being about treble its width. It was situated obliquely across the whole abdominal cavity. Its upper end was easily turned out of the abdomen, and the whole mass was lifted out of its bed. Its smooth, red surface nowhere indicated the outline of the uterus. Its lower end seemed to be one solid mass of pedicle extending completely across the whole inlet of the pelvis from side to side. The laminæ of the right broad ligament were separated in the most conspicuous manner that I ever beheld. The two ovaries were attached to the mass on a level with the umbilicus, having been lifted completely up into the abdominal cavity.

After button-holing the capsule of the tumor as low down as was practicable, it was peeled off as far as the finger could reach right and left, and ligatured in small masses in two places an inch or more apart and then divided between the ligatures. In this way all of the capsule that could be secured in ligatures and cut was soon treated, and the hæmorrhage from the operation was barely worth mentioning. The right uterine artery, which was much enlarged, was torn across and quickly secured. All of the spermatic and uterine arteries were secured with double ligatures of No. 14 silk. Thus the apparently large pedicle was much reduced in size and was found to be about equal to the normal *corpus uteri.*

Around this mass was placed a Kœberlé's *serre-nœud*, which was properly tightened, and the tumor excised. Small vessels were then secured. The clamp was then slowly loosened and the vessels of the stump were tied.

The cervical canal was conspicuously visible in the center of the stump. It was drawn up by a vulsellum as tautly as possible and excised for the length of at least one inch. The piece removed was cone-shaped, with its base upwards. Six stitches were then used, from side to side, placed so that when tightened the peritoneum was accurately brought together over the top of the stump. They were tied as tightly as they could be drawn. All further bleeding was attended to *secundam artem*, and the abdomen closed with nineteen stitches. The initial incision was made at 10:30 A.M., the stitches were begun at 11:42 A.M., and the operation was completed at 12:01 P.M.

She rallied from the operation in a few hours. For a period of four days afterwards she had a most intractable vomiting. After feeding by the rectum and permitting absolutely nothing to pass her lips, this troublesome symptom slowly diminished and gradually disappeared, but not until she had acquired a slightly pinched condition of the *alæ nasi*, which brandy caused to disappear. On the third day an offensive odor proceeded from the vagina. A carbolized solution permanently corrected it. It must have arisen from decomposition of the small amount of blood provoked by the introduction of the uterine sound.

Thereafter naught especially eventful occurred to direct attention to, excepting the following points :

1st. The patient never had an alvine dejection after the operation. Flatus passed only after the colonic distension permitted it, through a rectal tube. Repeated efforts were made to secure defecation, without result.

2d. At no time after the operation did she have a chill or sweats. The days were very hot and her greatest comfort was in being gently fanned and in dabbling her hands in a bowl of water placed at her side.

The following record of pulse, temperature and respiration was kept till the death of the patient, which occurred eleven days and nineteen hours post operation.

August 8.—5 P.M., P. 128, T. 101°, R. 18; 8 P.M., P. 120, T. 100.8°, R. 16; 10.45 P.M., P. 120, T. 100.2°, R. 20.

August 9.—8.45 A.M., P. 110°, T. 99.6, R. 20; 12.30 P.M., P. 116, T. 100°, R. 16; 8 P.M., P. 116, T. 100.3°, R. 24.

August 10.—10 A.M., P. 118, T. 100; 4 P.M., P. 112, T. 99.3°, R. 26; 8 P.M., P. 110, T. 99.5°, R. 22.

August 11.—8 A.M., P. 124, T. 100.5°, R. 22; 2 P.M., P. 110, T. 99.7, R. 18; 8.30 P.M., P. 128, T. 100.7°.

August 12.—8 A.M., P. 114, T.99.5°, R. 18; 2 P.M., P. 112, T. 98.7°, R. 17; 8 P.M., P. 114, T. 99.5°, R. 14.

August 13.—7 A.M., P. 114, T. 99.8°, R. 14; 4 P.M., P. 108, T. 99.6°, R. 14; 8 P.M., P. 114, T. 100.3°, R. 14.

August 14.—7 A.M., P. 112, T. 100.9°, R. 14; 2 P.M., P. 112, T. 99.8°, R. 16; 8 P.M., P. 116, T. 99.5°, R. 18.

August 15.—8 A.M., P. 120, T. 99.°, R. 16; 4 P.M., P. 116, T. 99.5°, R. 16; 8 P.M., P. 114, T. 99.4°, R. 16.

August 16.—8 A.M., P. 112, T. 100°, R. 14; 4 P.M., P. 108, T. 100.6°, R. 14; 10 P.M., P. 112, T. 100°, R. 16.

August 17.—8 A.M., P. 110, T. 99.4°, R. 16; 4 P.M.; P. 120, T. 99.6°, R. 16; 8 P.M., P. 120, T. 99.8°, R. 16.

August 18.—6 A.M., P. 118, T. 101.5°; 12.30 P.M., P. 114, T. 101.6°, R. 14; 8 P.M., P. 124, T. 102.8°, R. 18; 10 P.M., T. 102°; 11:50 P.M., T. 103.2°.

August 19.—1 A.M., T. 102°. Death at 6:45 A.M.

The autopsy was made thirty-two hours after death, and revealed a pelvis filled with fluid consisting of blood and pus. The small intestines were agglutinated over the pelvic inlet, almost hermetically. The surfaces of the small intestines turned towards the cavity of the pelvis presented a condition of sphacelus approaching demarcation. The pelvic cellular tissue was completely honeycombed with pus cavities. The stump presented a shrunken appearance, as though all of the stitches had been loosened, yet no pelvic fluid had found its way into the vagina through the cervical canal. The patient had succumbed to septicæmia. I regret that I did not drain.

Examination of the tumor revealed the fact that it was a multilocular fibroma, and that it grew from the anterior wall of the *corpus uteri*. The cavity of the uterus was found on a level with the umbilicus and contained a three months fœtus in unruptured membranes, evidently alive up to the time of operation. The cervical canal was 5½ inches long, which fact explains the immunity of the membranes from puncture by the introduction of a sound only four inches. The canal resembled the letter U, with its legs pulled apart, starting from the extreme left iliac fossa at the *os uteri* it passed towards the umbilicus two inches, and was then deflected at an obtuse angle towards the spleen.

Upon laying open the uterine cavity it was found to be in appearance a cavernous hollowing out of the posterior surface of the tumor. In front of it was nearly the whole width of the tumor. Its posterior boundary was a wall of uterine tissue thinner than the normal, unimpregnated posterior uterine wall. It was situated in the middle of the tumor.

The membranes presented a leaden, grayish appearance, and were filled with fluid, its smooth, undulating surface, in contrast with its nodular surroundings, looking not unlike the surface of "The Devil's Punch Bowl" on the top of Mt. Mangerton.

The fœtus was a male. Its cord was seven inches long and was normally attached.

Microscopic examination of the tumor by Dr. Ochsner shows its character to be purely *fibromatous*. Its weight was about ten pounds.

*Remarks.*—The removal of fibroid tumors which are slowly but surely killing patients has now passed into the category of recognized and justifiable operations. The astonishing successes of Keith in removing them are sufficient guaranty of justification for removing such growths under proper conditions.

But the complication of large fibrous tumors of the uterus with pregnancy presents the gravest possible condition for the surgeon's consideration. Conception seemed to give an impulse to the rapidity of the growth of the tumor under consideration that was very surprising. The arrest of its growth or its removal were the two horns of the dilemma. Against the production of abortion there are serious objections. It is followed by disproportionately great dangers and cannot possibly lessen the size of the growth materially.

The possibility of uncontrollable hæmorrhage from the seat of the placenta on a non-contractile mass of tumor must be faced in producing an abortion. In addition the dangers of septicæmia from puerperal disintegration of the tumor are not to be forgotten. While running the two risks of hæmorrhage and septicæmia from producing an abortion there is the very great possibility of avoiding a future extirpation of the whole mass by inducing a cessation of growth of the tumor, or even possibly by inducing a greater or lesser involution of the growth. The examination of the tumor shows conclusively that the uterus could never have extruded the fœtus and its adnexa through the elongated cervical canal because of the inutility of the uterine muscular fibre through fibromatous degeneration. Consequently abortion would in all probability have proven fatal through septicæmia. I am thoroughly convinced that the patient could not have lived many weeks longer without the death of the fœtus, when the overwhelming disaster of fatal sepsis would have speedily ensued without any compensatory explanation short of an autopsy, since the idea of pregnancy was about abandoned because abortion failed to occur; and the non-appearance of the fœtus *per vias naturales* would afford no satisfactory evidence of what had occurred, but, quite to the contrary, would have rendered a mysterious case much more mysterious. In such an event, *sine autopsia*, the pathological reasoning would have been that spontaneous necrosis of the fibroma had occurred and septicæmia followed.

*References.*—Hegar and Kaltenbach report six cases in the third edition of their "*Operative Gynecology*," 1886, as follows :

| Author. | Date. | Time of pregnancy. | Character of Tumor. | Result. |
|---|---|---|---|---|
| Kaltenbach | March 2, 1880 | Fifth month | Myoma | Recovery |
| Wasseige | March 18, 1880 | Fifth month | Myoma | Death 6th day |
| Nieberding | May 10, 1882 | Fourth month | ——— | Death 40 hours P. O. |
| Schroeder | June 10, 1884 | Third month | Myoma | Recovery |
| Schroeder | June 21, 1884 | Third month | ——— | Recovery |
| Walter | ——— 1883 | Fourth month | Colloid | Death 9th day |

CASE 7.—Dr. H. R. Storer amputated the pregnant uterus, in a primipara, æt 37 years, after three days of labor had passed, for a fibro-cystic tumor. The pedicle was constricted by a double metallic ligature and kept outside. The patient died in seventy hours of septicæmia. (*Journal of Gynecological Society of Boston*, October, 1869, p. 223.)

CASE 8.—Prof. S. Tarnier, Neuilly, France, on February 24, 1879, removed the uterus from a primipara, æt. 33 years, for a fibrous tumor of the uterus, after the patient had been in labor seven days. Condition of the patient at the time of operation very unfavorable. The fœtus was putrid, gas in the uterus having been found. Pedicle was kept out with a metallic pin and metallic ligature. Patient died of septicæmia on third day.. (*Annales de Gynécologie*, August, 1879, p. 81.)

CASE 9.—Dr. Zweifel, of Erlangen, Germany, on July 31, 1880, removed, from a primipara, æt. 37 years, the uterus, several hours after labor began, because of a fibroid tumor in the cervix. Patient died on the sixth day of septicæmia. The pedicle was tied with double silk ligature. (*Archiv. für Gynäkologie*, B. 17, H. 3.)

CASE 10.—Prof. Cataliatti, of Palermo, Italy, on October 28, 1880, removed the pregnant uterus from a primipara, æt. 41 years, for an interstitial fibroid of the posterior uterine wall soon after the inception of labor. The pedicle was kept outside with wire ligature and transfixed with metallic pin. Recovery followed. (*Bulletin dell. Academia di Medicina di Palermo*, 1880.)

CASE 11.—Dr. L. Prochownick, of Hamburg, Germany, performed hysterectomy, April 21, 1881, on a primipara, æt. 40 years, at seventh month, about twenty-four hours after the discharge of the liquor amnii, for a fibromyoma of the uterus impacted in the pelvis. The pedicle was held out of the abdomen with Péan's constrictor, two long pins, and stitched to the lower angle of the wound. The patient died in sixty hours p. o. from septicæmia. (*Deutsche Medicinische Wochenschrift*, No. 40, 1882.)

CASE 12.—Dr. Fochier, Lyons, France, on November 23, 1882, amputated the uterus at term, after the patient had been in labour three days, for a fibroid in the uterine cervix. Recovery followed. It was her fourteenth pregnancy. The pedicle was kept out in the lower angle of the wound. (*Lyon Méd.*, May 20, 1883.)

CASE 13.—Dr. M. Hanfield Jones reported in *Transactions of the Obstetrical Society of London*, vol. xvii, 1886, the case of a woman at term in whom delivery was impossible because of a subperitoneal fibroid in the true pelvis. The entire uterus and its appendages with the tumor was removed and the cervix clamped. The patient died of peritonitis on the third day.

CASE 14.—Amputation of the pregnant uterus at term, by Dr. M. Hofmeier, for fibroid tumor in the pelvic cavity. Reported by Dr. P. F. Mundé in the *American Journal of Obstetrics*, September, 1886, vol. xix, p. 905. Mother and child both saved.

CASE 15.—Large pediculated subperitoneal fibroid. Pregnancy, two months. Removal of tumor, uterus and ovaries.

Result not stated. Operator, Meredith. Reported in the *American Journal of Obstetrics*, September, 1886, p. 923.

PROFESSOR CHARLES T. PARKES read a paper entitled

II.—SUCCESSFUL REMOVAL OF THE UTERUS FOR FIBROIDS.

Mrs. L., an American lady, 37 years old, was first examined by me March 11, 1886. She had been married about nine years, but had never been pregnant. The consultation was held for the purpose of getting relief from an abdominal tumor, which first began to show evidence of its existence some three years ago. The first symptoms noticed was a burning pain in the right side, which continued with greater or less severity for about six months, when a small painless tumor was recognized deep in the right groin. This growth continued to enlarge steadily until she saw me, without any exacerbation of rapidity, until during the last six months, when it has grown more rapidly and its volume has increased to a greater extent than during the entire previous period of its history. Lately also the patient has lost rapidly in strength and flesh. There has been no interference with menstruation, that function being performed normally the entire time.

The tumor has seemed to diminish in size during menstruation, at least the feeling of fullness was lessened at those periods. The mammæ have not shown evidence of change of any nature. The right leg has swollen slightly for short periods of time and been the site of considerable neuralgic pain. On inspection the abdomen was evenly distended by a symmetrical tumor, occupying a central position and reaching close to the *appendix ensiformis;* the respiratory acts make no impression upon it. Palpation determined the presence of considerable free fluid in the peritoneal cavity and an indistinct, uncertain kind of fluctuation in the tumor itself. There was well-marked resonance surmounting the upper half of the tumor. The mass itself was quite tender to the touch. Vaginal examination revealed the os high in the pelvis, centrally located, with a small mass behind it, seemingly continuous with it, which was supposed to be the retroverted uterus. The free abdominal fluid rendered this examination unsatisfactory. No sound was passed. No positive diagnosis was made, but the supposition favored an ovarian growth. The patient desired an early operation.

She was advised to remain quietly in bed and to take such medicines as would probably lead to removal of the ascites. These instructions were carried out, and the second and final examination was made April 11, 1886. The patient was very much reduced in flesh and very anæmic, all the mucous surfaces exceedingly bloodless. Examination showed the peritoneal fluid entirely removed—the tumor standing out freely and prominently for inspection on all sides. It still seemed to give rise to fluctuation; palpated by several medical gentlemen, the testimony of all was that it contained fluid. To pressure the sensation was that of elasticity. Vaginal examination now showed plainly that the uterus was incorporated with the tumor; the sound went directly into it for five inches. On the posterior surface two small tumors were now plainly felt; the largest of them was supposed to be the retroverted uterus at the first examination. The diagnosis now was a uterine tumor.

The operation for removal was done at the Presbyterian Hospital, April 13, 1886, in the presence of Drs. Gunn,

Etheridge, Graham, Adolphus, Mitchell, Ochsner and others. An incision was made in the *linea alba* from the umbilicus to the pubes and the tumor exposed. It showed a white, glistening surface, very much like a cyst, and seemed to contain fluid. The introduction of the trocar was not followed by any flow of fluid, and during the manipulations the tumor changed to a darkish-red color. This change of color upon manipulation I have seen occur on other occasions, and its occurrence is a very sure indication of a uterine tumor. The incision was prolonged several inches above the umbilicus and the mass turned out of the cavity. After ligating and compressing the broad ligaments with forceps they were divided and the uterus cut through three-fourths of an inch above the cervico-vaginal junction. Paquelin's cautery was used to disinfect and destroy the cervical canal and to sear the uterine stump. It was also applied freely to the edges of the divided broad ligament on both sides. This controlled perfectly all hæmorrhage, except from two spurting arteries on the opposite lateral edges of the uterine stump; these were isolated and tied separately. All ligatures were of carbolized silk. A large rubber drainage-tube was carried to the bottom of Douglas's *cul-de-sac* and the extensive abdominal wound closed by eighteen sutures.

The patient was put to bed considerably collapsed, but rallied well, and as the record shows until she left for her home, May 30, never had a temperature of 100° F. There still remains a small sinus at the site of the drainage-tube, discharging about 1 dram of sero-pus in twenty-four hours. One of the silk ligatures has been discharged through it. The lady has gained flesh and strength rapidly, and is free from a trouble which was rapidly robbing her of all comfort in life and hastening her death.

The drainage-tube did excellent service in this case, allowing the exit of large quantities of bloody serum for many days after operation. In my practice its use has always served a good purpose when large raw surfaces were left in the abdominal cavity from whatever cause.

In spite of the absence of rise of temperature I have never managed an abdominal section in which the external wound did so badly. The main portions of the skin united by first intention, but a large abscess formed in the sheath of the rectus muscle, the pus from which extended from one end to the other of the wound; the nozzle of the irrigator introduced at one extremity of the wound and the carbolized water turned on, caused the pus to flow out freely from the opening at the lower end; the peritoneal edges united firmly at once and so saved trouble from this source.

It was slow in healing, several weeks, but finally united very soundly. The patient's very feeble vitality may account for the bad course taken by the wound. The wound was dressed, as I have been in the habit of doing for years, absolutely dry, with absorbent cotton and iodoform. My experience with these cases rather confirms a supposition that the frailest and weakest patients are the ones most likely to recover.

At the time of removal the tumor weighed fourteen pounds. Even after removal many gentlemen present were convinced from its consistence that it contained a cavity holding fluid, so very deceptive was its elasticity.

DISCUSSION.

PROFESSOR J. S. KNOX said : I want to call attention, in the case reported by Dr. Etheridge, to the rapid development of myomata after the establishment of pregnancy. I have two other cases of uterine fibroid that came under my care about the same time as the patient reported by Dr. Etheridge and who were treated similarly, and in whom also menstruation was not disturbed. They are still in perfect health, and the probabilities are that when they die it will be of some other disease ; one being a widow, the other an unmarried woman. The growth of this myoma before us was slow but persistent, in spite of the use of ergot, and until the time the menses ceased and pregnancy commenced, the woman enjoyed perfect health, all unfavorable symptoms appearing within three months preceding operation. Another fact I would like to call attention to is the danger of abortion in such cases. With the consent of Dr. Etheridge, I delayed any operative procedures until the woman had reached the full three months of pregnancy, that I might produce an abortion ; I introduced the sound with great difficulty, and passed it up full four inches ; it produced considerable hæmorrhage, which continued for about three days. The nausea ceased immediately and the patient noticed a diminution of the size of the breasts and lost the sense of fullness of the abdomen. Her appetite returned and she took a great deal of nourishment, which improved her physical condition very much: a strange result, seeing the abortion was not accomplished. In opening the tumor after its removal, I was satisfied that if I had produced the abortion the woman would probably have died of sepsis or hæmorrhage. There was no possible outlet for the contents of the uterus, and I think it was a fortunate thing that we did not succeed in accomplishing the abortion. Under similar circumstances I would prefer hysterectomy with its risks to abortion with its risks.

PROFESSOR E. C. DUDLEY said: Notwithstanding the general principle established by ovariotomy that the extra-peritoneal method is in itself dangerous, and that it should consequently be avoided whenever perfect intra-peritoneal hemostasis can be secured, and notwithstanding the fact that this general principle should stand for all operations involving the removal of abdominal tumors, yet the best statistics of the intra-peritoneal method in hysterectomy show a mortality of 30 or 40 per cent, while Keith and Bantock, with the clamp or *serre-nœud*, have a mortality of only 10 or 15 per cent. Now if it be true that certain dangers are inseparable from the extra-peritoneal method, it follows that part of this 10 or 15 per centum of mortality must be in consequence of it, and that the statistics might still further be improved if by some means an intra-peritoneal ligature could be applied which would give the same security against hæmorrhage and permit operators to dispense with the clamp. But the dangers of the silk ligature are greater than those of the clamp, because, however thoroughly applied, and however carefully the stump be stitched together, shrinkage of the stump from the escape of serum almost always occurs within a few hours, with consequent loosening of the ligatures and hæmorrhage, which may be fatal at once from great loss of blood, or later from septicæmia. The indication clearly is for an elastic ligature capable of following up the shrinking stump and of securing thereby permanent hemostasis. India rubber fulfills this indication, and if properly applied is capable of becoming readily and safely encysted. The experiments of Löwenhardt and Hallswachs, together with the numerous operations of Olshausen and others, give good evidence that the India-rubber tube when dropped into the abdomen is free from danger. It is true that certain cases have been reported in which sloughing of the pedicle has followed the application of the elastic ligature with resulting abscess; but this occurs in consequence of using a ligature of any material so large that it cannot imbed itself in the stump, and thereby permit the surface to close over it so as to secure the prompt establishment of collateral circulation to the distal portion of the stump.

Not very long ago the question was raised whether in supravaginal amputation it is necessary to remove the ovaries at the same time—a question to which experience has given an affirmative answer. Péan has observed a case of catamenial hæmatocele, Hegar a case of extra-uterine pregnancy and Kaltenbach a case of distressing molimen recurring monthly in consequence of leaving the ovaries after hysterectomy; therefore, unless the patient has passed the monopause, they should if possible be removed on principle.

DR. H. T. BYFORD said: I would like to ask whether such ligatures as Dr. Dudley speaks of could be prepared beforehand? [Dr. Dudley: You might prepare 300 of different lengths and select the one you want.] With such a [illegible]n tity of fresh ones on hand one could undoubtedly find one to use. Schroeder's later percentages are, I believe, a little more favorable than Dr. Dudley seems to think, and enable him to claim, with some degree of reason, that his intra-peritoneal method is the best.

THE PRESIDENT said: One word in relation to the remarks of Dr. Dudley concerning ligature: The shoemakers' stitch, using the silk ligature, was introduced in 1881, by Dr. Marcy, of Boston. I am not informed how many times it has been used. It comes nearer obviating the difficulties of the silk ligature, I think, than any other plan in use. His method is sewing the cervix with the shoemaker's stitch. One end of the silk ligature is carried through by a strong needle threaded at the point and mounted in a handle. This is then unthreaded and the other end threaded, bringing this through as the needle is withdrawn. In this way both ends of the suture lie in the same opening in the tissues, though running in opposite directions. Other stitches, as many as may be needed and as near together as is deemed necessary, are inserted in the same way.

This is a continuous suture, and consequently more elastic than the ordinary suture, and there is but one knot, at the termination of the suture, and if there seems to be danger from hæmorrhage a second row of sutures may be put in at right angles to the first. In the cases I have known with this suture there has been no difficulty from hæmorrhage or sepsis. The tumors were not large, and perhaps that explains the success of the method. I have never had an opportunity of trying it. It seems to me very desirable to use silk if we can to stop the hæmorrhage or oozing from the vessels. I don't exactly know Schroeder's plan of using the double row of stitches, whether it is substantially this method or not. One thing further which Dr. Merriman has touched upon, and which we should all profit by, viz.: that when there is evidence of sepsis, slight or considerable, we should open the abdominal wound. In all the autopsies I have made after these operations where sepsis has been the cause of death, I believe they could have been saved if the wound had been opened and properly treated three or four days before the death of the patient.

DR. H. P. MERRIMAN said: I was so fortunate as to be present at Dr. Etheridge's operation. One point that he did not mention is that after the removal of the tumor and after the use of the ligatures, which he applied to each artery by itself, and not in one mass around the pedicle, the pelvis was entirely dry, so much so that when the question of a drainage-tube came up, I believe every one present said they could see no reason for its use. There was no exudation. The sponge came out hardly colored after it was placed in contact with the surface that had been denuded, and there was apparently no need of drainage, yet afterward we had oozing from this surface and the formation of bloody serum in the cavity, with septicæmia as a result. I was present at the autopsy, and am convinced that had a drainage-tube been used this patient would have recovered. I don't know but that if after eight or nine days, when the symptoms began to show septicæmia, there had been an opening of the cavity, the septic material might have been removed and the case have had a fortunate termination. The danger seems to me to come from septicæmia rather than from any direct loss of blood there may be from hæmorrhage, although I can see that there might be, even with the drainage-tube, sufficient exudation of the parts to exhaust the patient and produce a fatal termination. There was one thing that struck me with considerable interest, and I don't know what bearing it may have. She had taken ergot uninterruptedly for a year with a general improvement of her health; after this use of the ergot she became pregnant for the first time in her eight years of married life. Was it a coincidence or was there some relation between the use of ergot and pregnancy?

PROFESSOR C. T. PARKES said: I am very much interested in this discussion, as it has brought up the question of how to treat the pedicle. Out of five hysterectomies that I have done, four by the intra-peritoneal and one by the extra-peritoneal method, the one that was treated by the elastic ligature died quicker than any of the rest. I am not favorably impressed with it, and think it is an unsafe ligature to use because it never ceases acting; it succeeds in stopping bleeding, but it is apt to stop all circulation, and in quite a number of these cases the portion beyond the ligature sloughs and you have a sloughing wound, and may have the slough lodging in the peritoneal cavity. In the case just referred to the stump of the uterus sloughed and was a stinking, decomposing mass on post-mortem examination. Another objection I have to this ligature is that it can be applied only to the uterus, the stump itself. There are many cases where, from one circumstance or another, you are led to operate and in which the tumor does not always have a pedicle that can be embraced by any ligature. The plan that I have used for a number of years in employing the elastic ligature for other purposes, is to draw it taut and tie the ends together with silk. By fastening the ligature while tightly drawn, you can cut the ends fairly short and have no knot at all. The plan I adopted in the operation reported tonight, was a combination of the silk ligature and the cautery. I believe it to be the best method. I was very much impressed on reading Dr. Keith's recent work on uterine tumors, by an expression in reference to the treatment of the pedicle. He now treats it almost entirely by the extra-peritoneal method, but thinks the intra-peritoneal will be the coming method, the pedicle being secured by some application of the cautery to the divided surfaces. This is the best method for controlling hæmorrhages. Often there will be certain spots where arteries of some size come up through the tissue, and perhaps an isolated ligature will have to be applied to them. I don't believe that any wound in the peritoneal cavity that is at all extensive in character, should be left without drainage. I have been satisfied, in the cases that have come under my observation, that one of the main causes of death has been from the neglect of this precaution. When I have divided a large surface, whether I have a dry cavity or not, if that surface is free in the peritoneal cavity, I shall use drainage. I do not think that it is possible to make any comparison between laparotomies for uterine tumors and ovarian tumors. There never will be the same conditions and the proportion of recoveries will never be the same. Dr. Keith does not pretend to say that one out of fifty of the cases that come to him are subjected to operation. I believe the proper method of controlling hæmorrhage and treating the stump in uterine tumors is by the combination of the silk ligature and searing the stump. The heat of the cautery I use is always a dark red. I have always adopted the plan of putting on to the stump that is to be seared the compress forceps or clamp, leaving a quarter of an inch of the tissue above it, and burning that down close to the forceps until it is perfectly smooth. I have always drained through the external wound, normally the abdominal cavity is closed and abhors any abnormal effusion; the elasticity of the walls and viscera presses it out if an opening be present.

PROFESSOR BARTLETT asked with reference to a case of double ovariotomy: At the time of the operation it was noticed that notwithstanding active purgatives had been used previously, the descending colon was full of hardened fæces; she subsequently developed obstruction of the bowel and it was impossible to get any motion at all. I examined the rectum high up, found a tight stricture, and got the bowels emptied after this was forcibly dilated.

